# Dynamic Transcriptome Sequencing of Bovine Alphaherpesvirus Type 1 and Host Cells Carried Out by a Multi-Technique Approach

**DOI:** 10.3389/fgene.2021.619056

**Published:** 2021-04-07

**Authors:** Dóra Tombácz, Norbert Moldován, Gábor Torma, Tibor Nagy, Ákos Hornyák, Zsolt Csabai, Gábor Gulyás, Miklós Boldogkői, Victoria A. Jefferson, Zoltán Zádori, Florencia Meyer, Zsolt Boldogkői

**Affiliations:** ^1^Department of Medical Biology, Faculty of Medicine, University of Szeged, Szeged, Hungary; ^2^Department of Biochemistry and Molecular Biology, Faculty of Medicine, University of Debrecen, Debrecen, Hungary; ^3^Institute for Veterinary Medical Research, Centre for Agricultural Research, Budapest, Hungary; ^4^Department of Biochemistry & Molecular Biology, Entomology & Plant Pathology, Mississippi State University, Starkville, MS, United States

**Keywords:** long-read sequencing technology, nanopore sequencing technology, synthetic long-read sequencing, herpesviruses, bovine herpesvirus, transcriptomics

## Introduction

Bovine alphaherpesvirus type 1 (BoHV-1) is an alphaherpesvirus causing a disease known as infectious bovine rhinotracheitis (Thiry et al., 2006), which causes serious economic losses in cattle industry (van Oirschot, 1995). As other alphaherpesviruses, e.g., pseudorabies virus (PRV), herpes simplex virus type 1 (HSV-1), and varicella zoster virus (VZV), BoHV-1 is also able to enter a latent state in the host's peripheral ganglia following primary infection (Jones, 1998), from which the virus can be reactivated (Nataraj et al., 1997). Bovine alphaherpesvirus type 1 has a large (~136 kbp) double-stranded DNA genome (d'Offay et al., 2013, 2016), which contains over 70 protein-coding and non-coding genes (Jefferson et al., 2018). Similar to other herpesviruses, BoHV-1 genes can be kinetically characterized as immediate-early (IE), early (E), and late (L) genes (L1: early-late; L2: “real” late), depending on the requirements for gene expression, and the stage of replication cycle they are expressed in (Harkness et al., 2014). The IE genes are transcription factors affecting the expression of other viral genes. E genes encode enzymes involved in DNA synthesis, whereas L genes specify structural components of virions. No well-annotated BoHV-1 transcriptome has been available until now (Glazov et al., 2010).

Short-read sequencing (SRS) technologies have revolutionized transcriptomics due to their capacity for massively parallel sequencing at a relatively low cost. In the last few years, long-read sequencing (LRS) has become an alternative methodology that is able to circumvent the limitations of SRS. In contrast to SRS, LRS techniques can efficiently detect transcript splice and length variants, and can distinguish overlapping transcripts. Long-read sequencing has recently been widely applied for transcriptome profiling in a variety of organisms (Byrne et al., 2017; Chen et al., 2017; Cheng et al., 2017; Moldován et al., 2017, 2018; Li et al., 2018; Nudelman et al., 2018; Tombácz et al., 2018a, 2020; Zhang et al., 2018; Boldogkői et al., 2019; Jiang et al., 2019; Viehweger et al., 2019; Zhao et al., 2019), including herpesviruses (Tombácz et al., 2015, 2016, 2017, 2018b; O'Grady et al., 2016; Balázs et al., 2017; Depledge et al., 2019). These approaches have disclosed much more complex transcriptional landscapes than formerly believed. LoopSeq, a new synthetic LRS technology, has been developed by Loop Genomics to present an alternative for the traditional LRS methodologies (Liu et al., 2020; Jeong et al., 2021; Nakatsuji et al., 2021). In short, LoopSeq leverages existing SRS approaches coupled to LoopSeq barcoding technology to enable long-read single-molecule sequencing on the Illumina platform. Reads with the same barcodes are grouped together and assembled into longer molecules using Loop Genomics' software. One of the major reasons to pursue the LoopSeq technology was its extremely low error rate compared to any other long read technologies. In addition, LoopSeq provides an independent approach which can be applied to confirm the results obtained by other techniques.

In this study, we generated time-varying datasets of the Cooper strain of BoHV-1 and host cell (bovine and ovine fibroblasts) transcriptomes using two LRS techniques. Samples were propagated in a Hungarian (ovine host) and in an American (Mississippi; bovine host) laboratory. Samples from mixed time points were sequenced from both batches with the aim to assess the effect of the viral infection on the host cell gene expressions. The infection time points from the Mississippian samples were also sequenced individually. Three biological replicates were used for the kinetic characterization of the viral and host cell RNA profiles. The Oxford Nanopore Technologies (ONT) amplified (1D) cDNA sequencing, direct cDNA (dcDNA), and direct RNA (dRNA) sequencing protocols [using oligo(dT) or random hexamer primers for reverse transcription], as well as the LoopSeq synthetic LRS approaches (Loop Genomics) were used to generate full-length transcripts. We were able to generate high quality long-reads by using these techniques for library preparations with the ONT MinION device, as well as the Illumina MiSeq sequencer.

Here, we provide a large genomic, as well as a static and a dynamic transcriptome catalog of BoHV-1. By applying state of the art genomic approaches, we were able to generate an expansive dataset that can be used for the identification of viral transcripts and RNA isoforms [including transcriptional start sites (TSS), transcriptional end sites (TES), and splice variations]. This transcriptomic data can also serve as a valuable resource for the kinetic characterization of the full-length viral transcriptome and for the profiling of virus–host cell interaction temporal dynamics. In addition to investigating RNA base modifications, various library preparation methods, the quality and length of sequencing reads derived from various sequencing approaches can also be compared with each other using our database. Furthermore, bioinformatics software, pipelines, and data analysis tools can be tested using this dataset.

## Methods

### Cells and Viruses

The Cooper strain of BoHV-1 was propagated in two different cell lines in two laboratories. Ovine kidney (OK) cells (ATCC CRL-6551) were used in a Hungarian laboratory (Institute for Veterinary Medical Research, Budapest, Hungary), while in the American laboratory (Mississippi State University, Starkville, Mississippi, USA), the Madin Darby Bovine Kidney (MDBK) cell line was used. Detailed information of the maintenance conditions is summarized in Supplementary Table 1A.

### Purification of Nucleic Acids

#### DNA From BoHV-1/MS

The supernatant of cells infected with BoHV-1/MS (propagated in the Mississippian laboratory) at an MOI (multiplicity of infection) of 5 was collected for 24 h, then centrifuged at 8,000 RPM for 30 min at 4°C to remove cellular debris. The pellet was discarded and the clarified supernatant was spun at 25,000 RPM in an ultracentrifuge for 2 h at 4°C with a 30% sucrose cushion to purify viral particles. The supernatant was decanted and the viral pellet was resuspended in TE buffer (1 M Tris-HCl, 0.2 M EDTA). Viral particles were lysed with a final concentration of 1% sodium dodecyl sulfate (SDS) at 37°C for 20 min before protein digestion with proteinase K (final concentration 0.5 mg/ml) at 60°C for 30 min. DNA was then extracted using phenol/chloroform/isoamyl alcohol mixture (25:24:1) at pH 8 (Acros Organics, Thermo Fisher Scientific). DNA was precipitated by its incubation in 3 M sodium acetate and 95% ethanol at −80°C for an hour. The DNA was then centrifuged at 14,000 RPM for 30 min at 4°C. The pellet was decanted and washed three times with 70% ethanol before one final centrifugation at 14,000 RPM for 15 min at 4°C. The supernatant was discarded and the pellet was suspended in TE buffer.

#### DNA From BoHV-1/HU

The supernatant of OK cells infected by BoHV-1/HU (propagated in the Hungarian laboratory) was centrifuged at 3,000 RPM for 5 min at 4°C. Virions were separated by ultracentrifugation at 23,500 RPM for 1 h at 4°C with a 30% sucrose cushion. Viral DNA was extracted using the Qiagen DNA easy Blood and Tissue Kit.

#### RNA

RNA from infected and mock-infected cells was purified using the NucleoSpin RNA kit (Machery-Nagel). In order to lyse cells, they were incubated in a buffer containing chaotropic ions, which inactivate RNases. Nucleic acid molecules were then bound to a silica membrane. All samples were treated with DNase I solution (from the kit) to remove residual genomic DNA contaminations. Total RNAs were eluted from the membrane in nuclease-free water. To eliminate any potential DNA contamination, samples were handled with TURBO DNA-free™ Kit (Invitrogen). The polyadenylated [Poly(A)+] RNA fraction of the total RNA was isolated using the Oligotex mRNA Mini Kit (Qiagen) in order to obtain the polyadenylated transcripts. For the investigation of the non-Poly(A)+ RNA molecules, rRNA depletion was carried out using Ribo-Zero Magnetic Kit H/M/R (Epicentre/Illumina).

### Library Preparation

#### Genome

Four micrograms of viral DNA was fragmented using covaris g-TUBE, at 4,200 RPM for 1 min. Three micrograms of DNA was used for ONT genomic DNA kit (SQK-LSK109). DNA strands were repaired by NEBNext FFPE DNA repair and Ultra II End-prep (New England Biolabs) enzymes. At the end of library preparation, adapter ligation was performed using NEBNext Quick T4 DNA Ligase. Between each step of the protocol the library was purified using Agencourt AMPure XP magnetic beads (Beckman Coulter). The two gDNA libraries were sequenced on two R9.4.1 Flongle Flow Cells (Oxford Nanopore Technologies).

#### Direct RNA Sequencing (dRNA-Seq) Using Oxford Nanopore Technologies—Mixed Time Points

Oxford Nanopore Technologies Direct RNA sequencing (DRS) protocol (Version: DRS_9080_v2_revL_14Aug2019) was used for amplification-free sequencing. Total RNAs from six time points [1, 2, 4, 6, 8, and 12 h post infection (p.i.)] were mixed together, then the PolyA(+) fraction was isolated from the mixture. The BoHV-1/HU sample [500 ng Poly(A)+ RNA] and the ONT Direct RNA Sequencing Kit (SQK-RNA001) were used for this experiment. RNA was mixed with oligo(d)T containing reverse transcription (RT) adapters (provided by the ONT kit), NEBNext Quick Ligation Reaction Buffer [New England Biolabs (NEB)], and T4 DNA Ligase (NEB). After 10 min of incubation at room temperature, dNTPs (10 mM, Thermo Fisher Scientific), the 5x first-strand buffer and DTT [both derived from the SuperScript IV Reverse Transcriptase kit (Thermo Fisher Scientific)] were added to the RT adapter-ligated RNA. SuperScript IV Reverse Transcriptase (Thermo Fisher Scientific) enzyme was added to generate the first cDNA strand. The RT was carried out at 50°C for 50 min, then the enzyme was inactivated at 70°C for 10 min. RNAClean XP beads (Beckman Coulter) were used to purify the sample. NEBNext Quick Ligation Reaction Buffer, RNA Adapter Mix, and T4 DNA Ligase were added to the reverse-transcribed RNA which were incubated for 10 min at room temperature. Lastly, the whole library (~200 ng) was loaded on a R9.4.1 SpotON Flow Cell (ONT).

#### Amplified cDNA Sequencing Using Oxford Nanopore Technologies—Mixed Time Points

Amplified cDNA libraries were generated from both the BoHV-1/HU and BoHV-1/MS samples using 1D Strand switching cDNA by ligation method (Version: DE_9063_v109_revK_14Aug2019) and the ONT Ligation Sequencing Kit 1D (SQK-LSK109) with oligo(dT) or random primers for the RT step. Fifty nanogams of Poly(A)+ RNA with poly(T)-containing primer (from the ONT Kit) or 50 ng rRNA-depleted RNA with custom made random hexamers were subjected to RT reactions. Ten millimolars of dNTPs were added to the RNA samples, then the mixture was incubated at 65°C for 5 min. Reverse transcriptions were carried out using SuperScript IV Reverse Transcriptase at 50 °C for 10 min. This was followed by the strand switching step at 42 °C for 10 min. Enzyme inactivation was performed at 80 °C for 10 min. KAPA HiFi DNA Polymerase (Kapa Biosystems) and Ligation Sequencing Kit Primer Mix (provided by the 1D Kit) were used to amplify the double-stranded (ds)cDNA. Supplementary Table 1B shows the PCR conditions. NEBNext End repair /dA-tailing Module (NEB) was used for repairing the cDNA ends, while the adapter (from the ONT Kit) ligation was executed with NEB Blunt/TA Ligase Master Mix (NEB). Agencourt AMPure XP magnetic beads were used for purification following each enzymatic step.

#### Synthetic Long-Read Sequencing on Illumina MiSeq Instrument

A synthetic LRS method was also applied for generating high quality sequencing reads. LoopSeqTM Transcriptome 3x8-plex Kit (Loop Genomics) was used for library preparation. The library was prepared from a multiplexed mixture of the 2 and 12 h p.i. samples in three biological replicates. Poly(A)+ RNA (diluted concentration: 0.1 ng/μl) samples were used as starting material and the phasing protocol of the Kit was used according to the manufacturer's recommendations. Briefly, the unique RT Primer (LoopSeq Kit), the diluted RNA sample, the BC RT Mix P, and the BC RT Enzyme P were mixed. This mixture was incubated as described in Supplementary Table 1C.

After a Post-barcoding SPRIselect Cleanup step (beads from the LoopSeq Kit), the cDNA samples were diluted 320-fold. Using reagents (Amplification Mix R and Amplification Additive) from the kit, the cDNA was amplified according to the protocol, summarized in Supplementary Table 1D. PCR products were purified with the SPRIselect Cleanup module of the LoopSeq Kit, then they were combined with the Distribution Mix and the Distribution Enzyme (Loop Genomics). The barcode distribution step was carried out at 20°C for 15 min, then the reaction was stopped by heating the reaction to 75°C for 5 min.

For barcode activation, Activation Mix and Activation Enzyme (provided by the LoopSeq Kit) were measured into the sample. These steps were carried out at 20°C for 16 h, then the reaction was heated to 65°C for 5 min. The barcodes were neutralized using the Neutralization Enzyme (37°C, 15 min). After a SPRIselect Cleanup step, the cDNA was fragmented using the Fragmentation Mix and the Fragmentation Enzyme from the Kit. These were added to the sample at 4°C. Next, the reaction was carried out at 32°C for 5 min. The enzyme was inactivated at 65°C for 30 min. This step was followed by end-repair and A-tailing using the Ligation Mix and the Ligation Enzyme at 20°C for 15 min. The sample was cleaned using the SPRIselect Cleanup reagents (part of the Kit). The cleaned sample was amplified with Index Master Mix and Index Primer P6 (sequence: GAATTCGT). The PCR protocol is shown in Supplementary Table 1E. The PCR product was purified with SPRIselect Cleanup reagents. The library (8 pM) was sequenced with an Illumina MiSeq Regent Kit v2 (300-cycle format). Paired-end sequencing was carried out. The cluster density was 1,145 cluster/mm^2^.

#### Time-Course Transcriptome Data Generation by ONT's Direct cDNA Sequencing Approach

Direct cDNA sequencing (dcDNA-Seq) was carried out from the mock infected sample and the six time points (1, 2, 4, 6, 8, and 12 h pi) from the BoHV-1/MS samples in three replicates using ONT's dcDNA Sequencing Kit (SQK-DCS109) according to the manufacturer's recommendations. In short, 100 ng Poly(A)+ RNA, the VN primer (from the ONT Kit) and 10 mM dNTP were mixed and incubated at 65°C for 5 min. This step was followed by the addition of 5x RT Buffer, RNaseOUT (Thermo Fisher Scientific), and Strand-Switching Primer (from the ONT Kit). This mixture was incubated at 42°C for 2 min, then Maxima H Minus Reverse Transcriptase was measured into the sample. Reverse transcription and strand-switching reactions were conducted at 42°C, 90 min. Next, the reactions were blocked by the heat inactivation of the enzyme (85°C, 5 min). The RNA was degraded from the RNA-cDNA hybrid using the RNase Cocktail Enzyme Mix (Thermo Fisher Scientific); the incubation temperature was 37°C for 10 min. The sample was purified (using AMPure XP beads), and then amplified using the LongAmp Taq Master Mix (NEB) and the PR2 Primer (from the ONT Kit). Supplementary Table 1F shows the PCR conditions. End-repair and dA-tailing of the fragmented DNA were carried out at 20°C for 5 min, using the NEBNext Ultra II End repair enzyme and buffer. The reaction was ended by heating the mixture to 65°C for 5 min. After an XP bead washing step, the sequencing Adapter Mix was ligated to the end-prepped cDNA with the Blunt/TA Ligation Master Mix (NEB; 10 min at room temperature). Finally, the adapted and tethered DNA library was purified and loaded into the R9.4.1 SpotON Flow Cells. Libraries were barcoded using Native Barcoding (12) Kit (ONT) following the manufacturer's recommendations. To avoid potential “barcode hopping,” mock-infected, 1 and 2 h p.i. samples were sequenced separately from the later time points.

### Pre-processing and Data Analysis

#### Genomic Datasets

**Figure 1:**
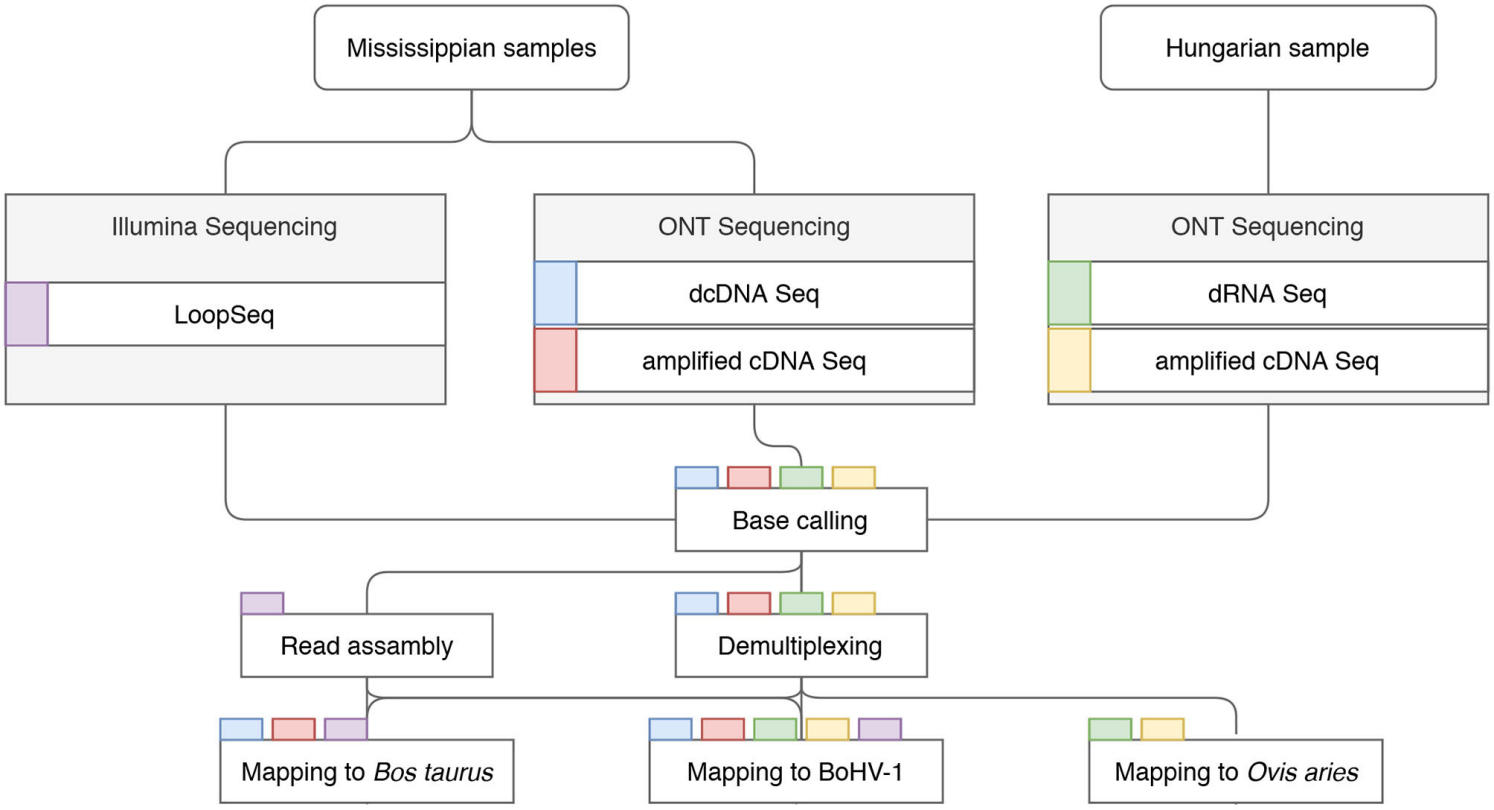
Detailed workflow of the project. Data flow chart shows the overview of the study design. Colored rectangles represent the sequencing libraries. Steps of data analysis are shown in rectangles, with the libraries undergoing the given step indicated by the tab color.

The MinION data was basecalled via the Guppy basecaller v. 3.4.1. software using—qscore_filtering. Reads with a Q-score >7 were assembled using the Flye software (Kolmogorov et al., 2019) with the—nano-raw-g 164k-m 1,000—meta options. The assemblies were aligned with the viral reference genome (JX898220.1) using Kalign (Lassmann and Sonnhammer, 2005). The ends of the BoHV-1/MS contig were trimmed, as the contig was assembled in a concatemer probably resulting from the sequencing of circularized genomic versions (Schynts et al., 2003). Discrepancies between the reference genome and the two assemblies were assessed manually using UGENE software suite (Okonechnikov et al., 2012). For data visualization we used IGV (Thorvaldsdóttir et al., 2013), WebLogo v.3.0 (Crooks et al., 2004), ComplexHeatmap (Gu et al., 2016), pheatmap_1.0.12, and ggplot2 (Wickham, 2009) R packages. For a schematic representation of our bioinformatics workflow see Figure 1.

#### Transcriptomic Datasets

The datasets from nanopore sequencing were basecalled using Guppy basecaller v. 3.4.1. with—qscore_filtering. Reads with a Q-score >7 were mapped to the circularized viral genome (NCBI nucleotide accession: JX898220.1) using Minimap2 aligner (Li, 2018). The same reads were also mapped to the host genomes, as follows: the BoHV-1/MS sample was mapped to the genome assembly of *Bos taurus* (GCF_002263795.1), while the BoHV-1/HU sample was mapped to the genome of *Ovis aries* (GCA_002742125.1) using Minimap2. Synthetic LRS data was basecalled on the Illumina MiSeq platform using Real-Time Analysis software with default settings. Basecalled reads were then processed by the Loop Genomics Pipeline Software with default settings. Synthetic long reads were mapped to the BoHV-1 and *Bos taurus* genomes using Minimap2 software. The aligned reads are available at FigShare as BED (Browser Extensible Data) files and can be viewed by Geneious (Supplementary Note 1).

### Code Availability

**Guppy v3.4.5:** https://community.nanoporetech.com/downloads?fbclid=IwAR2IchRL4gDnfA6h996UkN4vS5pbB_u6rUtKVFX3aTiBHsWFknglQ6FyvPkg

**minimap2:** https://github.com/lh3/minimap2

**STAR:** https://github.com/alexdobin/STAR

**samtools:** https://github.com/samtools/samtools

**SeqTools**: https://github.com/moldovannorbert/seqtools

## Sequencing Yield Report

**Figure 2:**
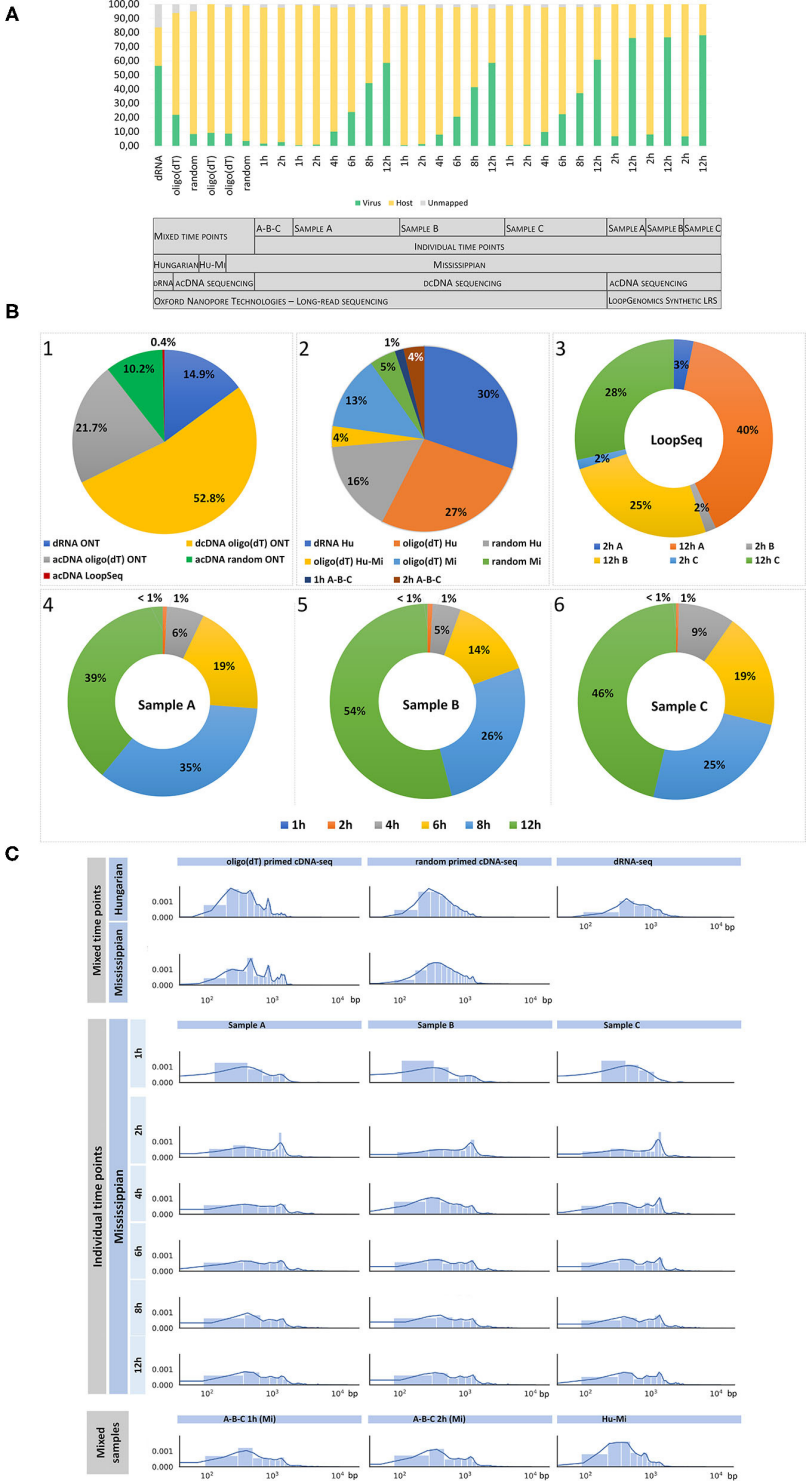
Summary statistics of the dataset. This figure shows the statistics of sequencing reads, including the virus:host read ratios **(A)**, the proportion between the total viral reads **(B1)** generated by various sequencing techniques, and the ratios of reads obtained from non-time-varying (“static”) samples **(B2)** and time-varying (“dynamic”) samples **(B3–6)**, which were generated by nanopore sequencing **(B2, B4, B5, B6)** or by LoopSeq **(B3) (C)** Read length distribution in the various samples. **(A)** Virus:host ratio of transcription reads. This study yielded altogether 3,463,245 viral and 22,337,732 host transcript reads. The first six samples were prepared by mixing RNAs isolated from time-varying samples before library preparation using equal volumes of RNA solution taken at each time point (1, 2, 4, 6, 8, and 12 h post infection; “mixed time points” in the gray chart). “A-B-C” in the gray chart include those 1 and 2 h samples, which contain combined data of the three biological replicates. The rest of the bars depict the virus:host ratio of time-course data obtained from three sample series (A–C, each representing a biological replicate) which were generated using nanopore sequencing (samples from time points 1, 2, 4, 6, 8, and 12 h), or via LoopSeq (samples from time points 2 and 12 h). Reverse transcription was carried out using oligo(dT) oligonucleotide primers in each case, except in two samples where random oligonucleotide primers were used (labeled as “random”). Some of the cDNA libraries were generated using PCR amplification (labeled as “acDNA,” where “a” stands for “amplified”), the rest of the cDNA samples were non-amplified (labeled as “dcDNA,” where “d” stands for “direct”). “Hu-Mi” refers to the mixed time points from the Hungarian and Mississippian samples, which were sequenced using barcode labeling. A small ratio of the barcode sequences had lower sequencing quality. These reads were not excluded because they can be used for further analysis of the virus. LRS: long-read sequencing. **(B)** Viral read ratios in the different samples. The first pie chart diagram (B1) shows the relative proportion of the total viral reads generated by the various sequencing approaches. More than half of the viral reads were produced by direct cDNA (dcDNA) sequencing technique, whereas 0.4% of the total reads were generated by the LoopSeq technique. The second pie chart diagram (B2) illustrates the proportions of non-time-varying (“static”) transcript reads obtained by nanopore sequencing. Charts B3–B6 represent the relative proportions of time-varying (“dynamic”) reads generated by LoopSeq (B3) or nanopore dcDNA sequencing (B4–6) at different time points. The ratios of the three biological replicates (samples A–C) of the LoopSeq data (2 and 12 h samples) are depicted in a single chart, while the nanopore data (1, 2, 4, 6, 8, and 12 h samples) were illustrated in three charts, each representing one of the three biological replicates. **(C)** Length distribution of sequencing reads aligned to the BoHV-1 genome. The shortest reads were generated by direct RNA sequencing (dRNA-Seq; mean of viral reads: 970 bp), whereas the dcDNA technique yielded the longest reads (ranging between 1,202 and 1,846 bp in the different samples).

The comprehensive workflow of the study is shown in Figure 1. ONT sequencing produced altogether 22,337,732 reads mapping to the host genomes and 3,450,627 sequencing reads mapping to the BoHV-1 genome (read statistics are illustrated in Figure 2). LoopSeq analysis generated 36,627,542 sequencing reads, which were assembled into 27,098 contigs representing full-length reads. Of these, 12,618 mapped to the viral genome. The detailed summary statistics is presented in Supplementary Tables 2A,B. We carried out transcriptome sequencing of the two cell lines on which the viruses were propagated. A total of 17,648,405 MinION reads and 14,671 assembled LoopSeq Illumina reads mapped to the *Bos taurus* genome, whereas 4,674,656 MinION reads mapped to the *Ovis aries* genome. Detailed summary statistics can be found in Supplementary Tables 2C–E. The global virus:host ratio of transcription reads is 13.4% in our entire dataset. Figure 2A shows the values of these ratios in the different samples. The exact ratio in each sample is dependent on the titer of the virus used for the infection and on the stage of the viral life cycle at the examined time point. Furthermore, the applied sequencing approach also affects the virus to host ratio of read counts because e.g., the efficiency of random oligonucleotide-primed reverse transcription is lower for the very high G/C containing BoHV-1 genome than for host genomes. A small proportion of reads is not mapped to either the virus or the host genome. The reason for this may be poor read quality. The ratios of viral reads generated by the different sequencing approaches are shown in Figure 2B. It should be noted that the large difference between the yields of the sequencing approaches is not caused by technical differences but by the number of libraries and flow cells used for the given sequencing methods. Figure 2C shows the average read length produced by the various sequencing techniques. The quality of barcode sequences on many reads had lower quality, therefore the Guppy basecaller was unable to demultiplex them. However, these samples can be used to analyze the structural variants of viral and host transcripts.

## Technical Validation and Data Re-Use

The quantity of the samples was determined using Invitrogen Qubit 4 Fluorometer (Thermo Fisher Scientific) with Qubit RNA BR Assay Kit, Qubit RNA HS Assay Kit, or dsDNA HS Assay Kit (Thermo Fisher Scientific). The quality of the RNA samples and libraries were checked using Agilent's TapeStation 4150. RNAs with RIN scores above 9.6 were used for cDNA production. For the analysis of virus–host interactions three biological replicates were sequenced from each individual time point.

The present work generated a dataset using cutting-edge sequencing technologies (ONT MinION and Illumina MiSeq platforms). We applied several library preparation approaches, including synthetic LRS for the first time in viral transcriptome research. These data allow the kinetic analysis of the full-length viral transcriptome, as well as of the MDBK cell line. The primary aim of this study was to provide a large dataset for the kinetic analysis of the BoHV-1 transcriptome and to determine its complexity. An additional goal was to generate a deep coverage, long-read dataset for the analysis of the different transcript isoforms, including length (TSS and TES) variants; mono-, and polycistronic, as well as complex transcripts; and to define full-length viral RNA molecules. The dataset can also be useful for examining the effect of the viral infection on the host transcriptome.

The provided BAM files contain reads already mapped to the virus and to the host genomes (Figure S1). These aligned reads can be further analyzed by comparing them to the results of various long-read aligners [e.g., NGMLR (Sedlazeck et al., 2018)], bioinformatics tools [e.g., samtools (Li et al., 2009), or bedtools (Quinlan and Hall, 2010)]. Other software or pipelines for LRS data analysis [e.g., SQANTI (Tardaguila et al., 2018) or LoRTIA] and visualization tools Geneious (Kearse et al., 2012), Artemis (Rutherford et al., 2000), IGV (Robinson et al., 2011), or CLC Genomics Workbench can be tested using this dataset.

The quality and yield of the applied nanopore and synthetic LRS techniques and the various ONT library preparation approaches can also be compared to one another. The provided dataset could be used to better understanding the general principle of gene expression control of alpha-herpesviruses.

## Data Availability Statement

The datasets generated for this study can be found in online repositories. The names of the repository/repositories and accession number(s) can be found below: https://www.ebi.ac.uk/ena, PRJEB33511.

## Author Contributions

DT and ZC carried out the library preparation, sequencing, and participated in the data analysis. AH, VJ, and FM propagated the viruses. NM, GT, GG, TN, and MB carried out the bioinformatic analysis. DT and GT generated the Figures. ZB conceived and designed the study. FM participated in the design of the methodology. ZB and DT wrote the manuscript with feedback from all co-authors. All authors contributed to the article and approved the submitted version.

## Conflict of Interest

The authors declare that the research was conducted in the absence of any commercial or financial relationships that could be construed as a potential conflict of interest.
