# Supplementary material for: Dynamic Transcriptome Sequencing of Bovine Alphaherpesvirus Type 1 and Host Cells Carried Out by a Multi-Technique Approach
Source: Front Genet. 2021 Apr 7;12:619056. doi: 10.3389/fgene.2021.619056 (PMC8059770; doi:10.3389/fgene.2021.619056)
Supplement: Supplementary file 4 [file Data_Sheet_1.docx]

Supplementary Material

# Supplementary Figures, Tables and Note

## Supplementary Figures


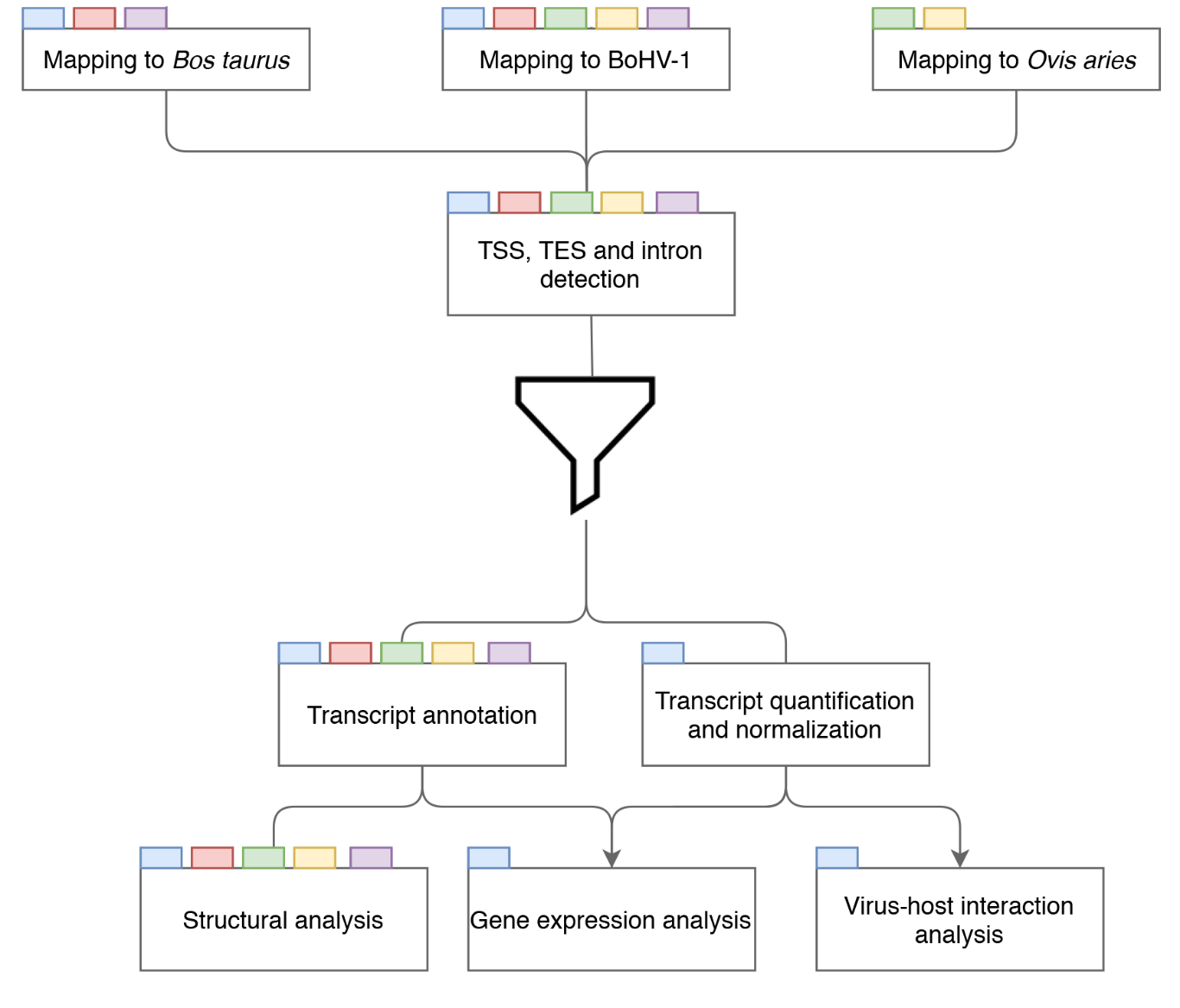


**Supplementary Figure 1. Schematic representation of the bioinformatics workflow and the potential re-usage of the dataset.** Colored rectangles represent the sequencing libraries (as in Figure 1). Steps of data analysis are shown in rectangles, with the libraries undergoing the given step indicated by the tab color. The funnel symbol represents the recommended TSS, TES and intron filtering.

## Supplementary Tables

**Supplementary Table 1. Summary tables of reaction conditions.**

**A.** Comparison of the cell culturing conditions which were used in this study. **B.** PCR settings which were used for the amplification of ONT cDNA-seq libraries. **C.** Reverse transcription incubation temperatures and time used for LoopSeq library preparation. **D.** PCR amplification conditions which were applied for LoopSeq library generation during the 1st PCR round. **E.** PCR amplification conditions which were applied for LoopSeq library generation during the 2nd PCR round. **F.** ONT’s direct cDNA-sequencing conditions for PCR.

**Supplementary Table 2. Summary tables.**

**A.** Statistics of ONT MinION sequencing reads mapped to the viral genome. **B.** Statistics of synthetic long-read sequencing reads mapped to the viral genome. **C.** Statistics of ONT MinION sequencing reads mapped to the host (*Bos taurus*) genome. **D.** Statistics of synthetic long-read sequencing reads mapped to the host (*Bos taurus*) genome. **E.** Statistics of ONT MinION sequencing reads mapped to the host (*Ovis aries*) genome.

**Supplementary Table 3. Metadata sheet of the sequencing dataset.**

The metadata describes the technical aspects of sequencing experiments, namely sequencing libraries, preparation techniques and data files. Abbreviations: ONT – Oxford Nanopore Technologies; dRNA – direct RNA sequencing, dcDNA – direct cDNA sequencing; 1D cDNA – amplified cDNA sequencing.

**1.3 Supplementary Note 1.** **Information for downloading and using the Geneious file.**

The whole raw dataset (reads) mapped to BoHV-1, *Bos Taurus* and *Ovis aries* genomes, are available at FigShare: 10.6084/m9.figshare.14124998. The dataset can be view by using Geneious software which is available at <https://www.geneious.com>

|  |  |  |  |  |  |  |  |  |  |  |  |  |  |  |  |  |  |  |  |  |
| --- | --- | --- | --- | --- | --- | --- | --- | --- | --- | --- | --- | --- | --- | --- | --- | --- | --- | --- | --- | --- |
|  |  |  |  |  |  |  |  |  |  |  |  |  |  |  |  |  |  |  |  |  |
|  |  |  |  |  |  |  |  |  |  |  |  |  |  |  |  |  |  |  |  |  |
|  |  |  |  |  |  |  |  |  |  |  |  |  |  |  |  |  |  |  |  |  |
|  |  |  |  |  |  |  |  |  |  |  |  |  |  |  |  |  |  |  |  |  |
|  |  |  |  |  |  |  |  |  |  |  |  |  |  |  |  |  |  |  |  |  |
|  |  |  |  |  |  |  |  |  |  |  |  |  |  |  |  |  |  |  |  |  |
|  |  |  |  |  |  |  |  |  |  |  |  |  |  |  |  |  |  |  |  |  |
|  |  |  |  |  |  |  |  |  |  |  |  |  |  |  |  |  |  |  |  |  |
|  |  |  |  |  |  |  |  |  |  |  |  |  |  |  |  |  |  |  |  |  |
|  |  |  |  |  |  |  |  |  |  |  |  |  |  |  |  |  |  |  |  |  |
